# Interface Superconductivity in a Dirac Semimetal NiTe_2_

**DOI:** 10.3390/nano12234114

**Published:** 2022-11-22

**Authors:** Varnava D. Esin, Oleg O. Shvetsov, Anna V. Timonina, Nikolai N. Kolesnikov, Eduard V. Deviatov

**Affiliations:** Institute of Solid State Physics of the Russian Academy of Sciences, Moscow District, 2 Academician Ossipyan Str., 142432 Chernogolovka, Russia

**Keywords:** topological semimetals, Dirac materials, superconductivity

## Abstract

We experimentally investigated charge transport through a single planar junction between a NiTe${}_{2}$ Dirac semimetal and a normal gold lead. At milli-Kelvin temperatures, we observe non-Ohmic dV/dI(V) behavior resembling Andreev reflection at a superconductor–normal metal interface, while NiTe${}_{2}$ bulk remains non-superconducting. The conclusion on superconductivity is also supported by the suppression of the effect by temperature and magnetic field. In analogy with the known results for Cd${}_{3}$As${}_{2}$ Dirac semimetal, we connect this behavior with interfacial superconductivity due to the flat-band formation at the Au-NiTe${}_{2}$ interface. Since the flat-band and topological surface states are closely connected, the claim on the flat-band-induced superconductivity is also supported by the Josephson current through the topological surface states on the pristine NiTe${}_{2}$ surface. We demonstrate the pronounced Josephson diode effect, which results from the momentum shift of the topological surface states of NiTe${}_{2}$ under an in-plane magnetic field.

## 1. Introduction

A search for topological superconductivity is one of the topics that supports an interest in Dirac materials. As the most famous example, superconductivity has been observed in twisted bilayer graphene [1,2,3]. Flat-band formation is considered as the favorite explanation for these intriguing results [4,5,6,7]. For the stacking of graphene layers, the nodal line is formed in the bulk, which is the source of the topological protection of the surface band. The two-dimensional surface flat-bands are formed from the zero energy states on the top and bottom surfaces of this artificial nodal line semimetal. The boundaries of the flat bands are the projections of the nodal loop in bulk to the top and bottom surfaces. In the presence of attractive interaction due to electron–phonon coupling, the extremely singular density of states associated with the flat band dramatically increases the superconducting transition temperature [8,9].

Apart from graphene, topological semimetals are also characterized by Dirac spectrum. The previous considerations on the surface flat-band can be naturally extended for nodal-line semimetals [8,9]. However, they are also valid for other topological semimetals [10], since the flat-band and topological surface states are closely connected: as supported by the topological-insulator-multilayer model, the stacking of layers of topological insulator leads to formation of a semimetal with Fermi arc surface states [11], so the flat-band is the topological surface state at zero energy [5,9]. The flat band remains even if the nodal line extends, reaches the boundaries of the Brillouin zone and disappears there. In this case, the nodal line semimetal transforms into the 3D topological insulator, and the surface flat bands are extended to the whole 2D Brillouin zones on the top and bottom surfaces [8].

For the Cd${}_{3}$As${}_{2}$ Dirac semimetal, flat bands are evidenced in angle-resolved photoemission spectroscopy (ARPES) [12,13] and magneto-optic [14,15] experiments. The surface superconductivity was experimentally observed in direct transport experiments [16,17]. Furthermore, the point contact spectroscopy experiments [18,19] reveal the signatures of superconductivity in a tip contact region (so-called tip-induced superconductivity), while the pressure of a tip is obviously not enough to induce bulk superconductivity [20] in Cd${}_{3}$As${}_{2}$. Due to the different experimental techniques, these results require a general explanation, so the flat-band-stimulated superconductivity approach has the advantage of its independence of the experimental details.

Due to the topological origin, the effect should be also independent on the particular material. NiTe${}_{2}$ is a recently discovered Dirac semimetal belonging to the family of transition metal dichalcogenides. The nontrivial topology of NiTe${}_{2}$ single crystals was confirmed by spin-resolved ARPES [21,22].

Though bulk NiTe${}_{2}$ single crystals have finite resistivity down to mK temperature, the effect of the topological surface states on superconductivity was demonstrated as the Josephson diode effect [23]. In general, Cooper pairs can acquire a finite momentum and give rise to a diode effect in superconductors with strong spin-orbit coupling [24,25,26]. In NiTe${}_{2}$, the finite momentum pairing results from the momentum shift of the topological surface states under an in-plane magnetic field due to the spin-momentum locking, as confirmed by the ARPES measurements [23]. Since the flat-band and topological surface states are closely connected, it is also reasonable to search for the interface superconductivity in this type-II Dirac semimetal.

Here, we experimentally investigate the charge transport through a single planar junction between a NiTe${}_{2}$ Dirac semimetal and a normal gold lead. At milli-Kelvin temperatures, we observe non-Ohmic dV/dI(V) behavior resembling Andreev reflection at a superconductor–normal metal interface, while the NiTe${}_{2}$ bulk remains non-superconducting. In analogy with the known results for Cd${}_{3}$As${}_{2}$ Dirac semimetal, we connect this behavior with interfacial superconductivity due to the flat-band formation at the Au-NiTe${}_{2}$ interface. Since the flat-band and topological surface states are closely connected, the claim on the flat-band-induced superconductivity is also supported by the pronounced Josephson diode effect on the pristine NiTe${}_{2}$ surface.

## 2. Materials and Methods

NiTe${}_{2}$ was synthesized by elements which were taken in the form of foil (Ni) and pellets (Te). The mixture was heated in an evacuated silica ampule up to 815 °C with the rate of 20 deg/h, and the ampule was kept at this temperature for 48 h. The layered single crystal was grown in the same ampule by the gradient freezing technique with the cooling rate of 10 deg/h. The obtained crystal was of 80 mm length and 5 mm thickness.

**Figure 1:**
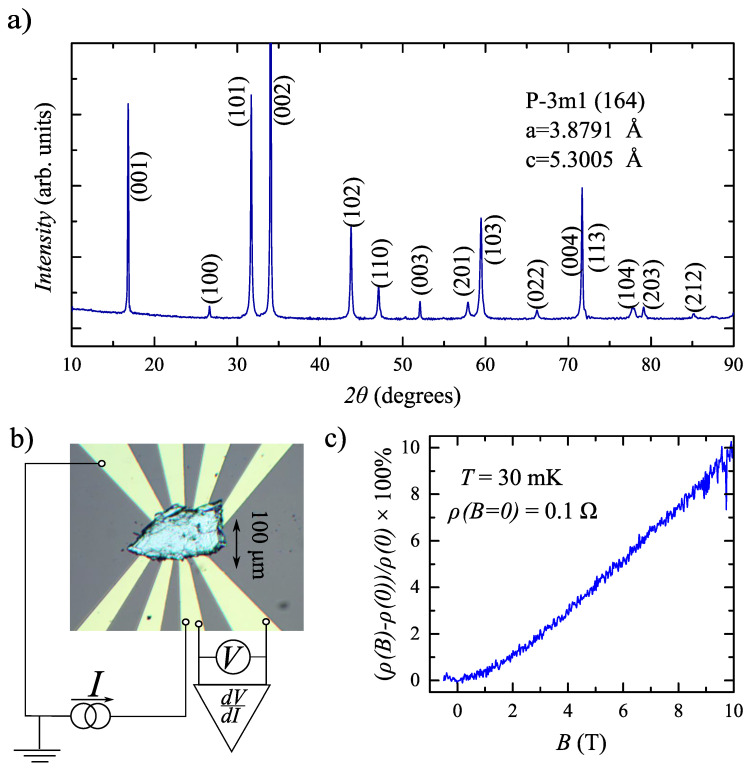
(Color online): (**a**) X-ray powder diffraction pattern, which confirms a single-phase NiTe${}_{2}$ with P-3m1 (164) space group (a = b = 3.8791 Å, c = 5.3005 Å). (**b**) A top-view image of the sample with Au leads. A thick (0.5 μm) NiTe${}_{2}$ mechanically exfoliated flake is placed on the pre-defined Au leads pattern to form 5 μm separated NiTe${}_{2}$-Au junctions with a lateral size of approximately 10 μm. To investigate a single NiTe${}_{2}$-Au junction, electron transport is measured by a standard three-point technique. (**c**) The known non-saturating magnetoresistance for NiTe${}_{2}$ material [33,34] is reproduced for our samples in four-point longitudinal magnetoresistance measurements. The four-point resistance is finite (≈0.1 Ω) even in the zero field. The data are obtained at *T* = 30 mK for the normal magnetic field orientation.

A nearly stoichiometric ratio Ni${}_{1-x}$Te${}_{2}$ (*x* < 0.06) is verified by the energy-dispersive X-ray spectroscopy. The powder X-ray diffraction analysis (Cu Kα1 radiation, λ = 1.540598 Å) confirms the single-phase NiTe${}_{2}$ with P-3m1 (164) space group (a = b = 3.8791 Å, c = 5.3005 Å), as can be see Figure 1a. The known structure model is also refined with single-crystal X-ray diffraction measurements (Oxford diffraction Gemini-A, Mo Kα), and the crystal cleavage plane is verified to have (0001) orientation.

Figure 1b shows a top-view image of a sample. Despite the fact that NiTe${}_{2}$ can be thinned down to two-dimensional monolayer samples, topological semimetals are essentially three-dimensional objects [11]. Thus, we have to select relatively thick (above 0.5 μm) NiTe${}_{2}$ single crystal flakes, which also ensure sample homogeneity. Thick flakes require a special contact preparation technique: the fresh mechanically exfoliated flake is transferred onto the Au leads pattern, which is defined on the standard oxidized silicon substrate by lift-off technique, as depicted in Figure 1b. The transferred flake is shortly pressed to the leads by another oxidized silicon substrate, and the latter is removed afterward. The 100 nm-thick, 10 μm-wide Au leads are separated by 5 μm intervals under the flake. This procedure provides transparent Au-NiTe${}_{2}$ junctions (approximately 2 Ohm resistance for the 10 μm lateral size), stable in different cooling cycles, which was been verified before for a wide range of materials [16,27,28,29,30,31,32]. As an additional advantage, the obtained Au-NiTe${}_{2}$ junctions are protected from any contamination by the SiO${}_{2}$ substrate.

The quality of our NiTe${}_{2}$ material can be tested in standard four-point magnetoresistance measurements, as can be seen in Figure 1c. NiTe${}_{2}$ flakes demonstrate the non-saturating longitudinal magnetoresistance for the normal magnetic field orientation, which effectively reproduces the previously reported one for this material [33,34]. The four-point resistance is finite (0.1 Ω) between 5 μm-spaced Au leads, so there is no superconductivity for bulk NiTe${}_{2}$ single-crystal flakes at ambient pressure. The measurements are performed within the 30 mK–1.2 K temperature range in a dilution refrigerator equipped with a superconducting solenoid.

## 3. Results

### 3.1. Single Au-NiTe${}_{2}$ Junctions

**Figure 2:**
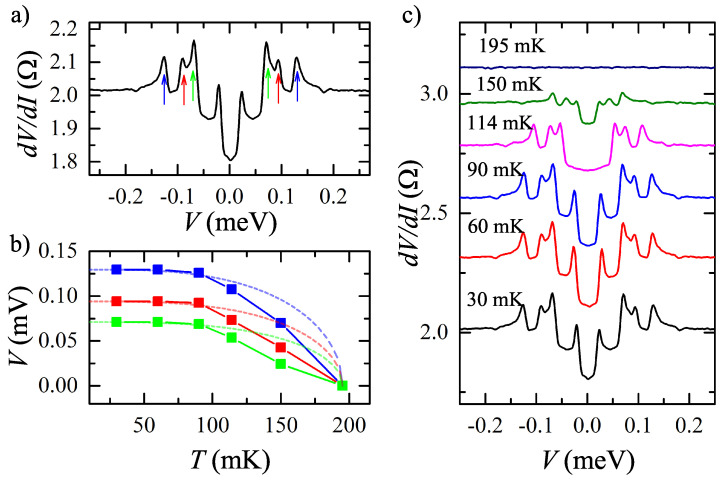
(Color online) (**a**) Non-Ohmic behavior of dV/dI(V) differential resistance for a single Au-NiTe${}_{2}$ junction at 30 mK temperature in the zero magnetic field. The dV/dI(V) curve shows a prominent dV/dI drop around the zero bias, which is accompanied by several dV/dI peaks at higher biases (denoted by arrows for the positive bias polarity). (**b**) Temperature dependence of the peaks’ positions. The colors correspond to the arrow colors in the (**a**) panel. The standard BCS fit [35] is shown by the dashed lines. (**c**) Temperature dependence of the dV/dI(V) curves in the zero magnetic field. The central dV/dI drop is diminishing with the temperature, while dV/dI peaks’ positions also move to the zero bias. The curves are shifted for clarity.

We study electron transport across a single Au-NiTe${}_{2}$ junction in a standard three-point technique: one Au contact is grounded and two other contacts are used as current and voltage probes, as schematically presented in Figure 1b. To obtain dV/dI(V) characteristics in Figure 2a, the dc current is additionally modulated by a low (100 nA) ac component. We measure both dc (*V*) and ac (∼dV/dI) voltage components with a dc voltmeter and a lock-in amplifier, respectively. The signal is confirmed to be independent of the modulation frequency within the 100 Hz–10 kHz range, which is defined by the applied filters.

Figure 2a shows non-Ohmic behavior of dV/dI(V) differential resistance for a single Au-NiTe${}_{2}$ junction. We observe the prominent (approximately 10%) dV/dI drop around the zero bias, which is accompanied by several dV/dI peaks at higher biases. For a fixed grounded Au contact, the dV/dI(V) curve is verified to be independent of mutual positions of current/voltage probes, so it mostly reflects the resistance of the Au-NiTe${}_{2}$ interface (approximately 2 Ω) without noticeable admixture of the sample’s bulk (≈0.1 Ω resistance in Figure 1c).

Figure 2b,c shows a dV/dI(V) temperature dependence. The non-Ohmic behavior can only be seen below the $T_{c}\approx$ 190 mK critical temperature. The central dV/dI drop is diminishing with the temperature, while dV/dI peaks’ positions also move to the zero bias. The latter dependence is directly depicted in Figure 2b for three successive peaks from Figure 2a.

**Figure 3:**
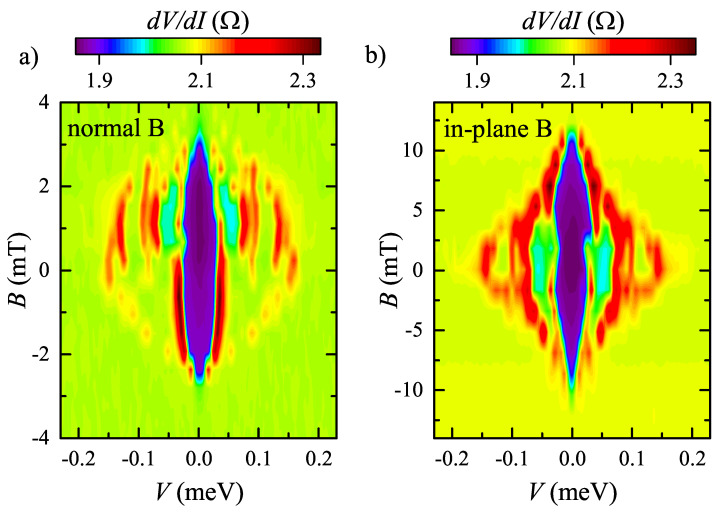
(Color online) Suppression of dV/dI(V) non-Ohmic behavior by magnetic field at 30 mK temperature for normal (**a**) and in-plane (**b**) field orientations. The behavior is qualitatively similar, while the critical fields $B_{c}$ differ significantly for these two orientations. There is also some asymmetry of the colormap for normal field orientation (**a**), which is verified to be independent on the magnetic field sweep direction.

Non-Ohmic behavior can also be suppressed by magnetic field, as can be seen in Figure 3a,b for normal and in-plane field orientations, respectively. Despite the qualitative similarity, the critical fields differ significantly for these two orientations: $B_{c}$ can be estimated as 3 mT in Figure 3a and as 10 mT in Figure 3b. We also observe some asymmetry of the colormap in Figure 3a for normal magnetic field, which cannot be seen for in-plane orientation in Figure 3b. The normal-field asymmetry is verified to be independent on the magnetic field sweep direction. The asymmetry cannot be connected with magnetic ordering in the bulk of NiTe${}_{2}$, since the magnetometry revealed a purely paramagnetic susceptibility for our NiTe${}_{2}$ crystals in accordance with the previously reported data [33].

**Figure 4:**
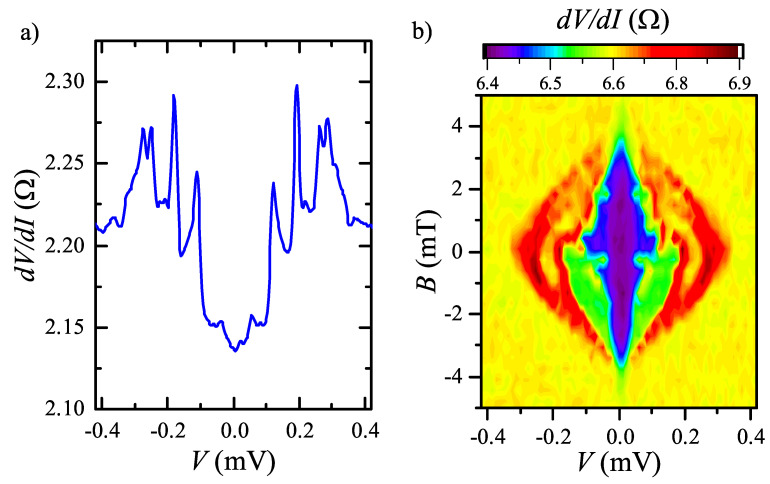
(Color online) (**a**) Non-Ohmic behavior of dV/dI(V) for a different sample to show typical sample-to-sample variation. The behavior is qualitatively the same as for the first junction in Figure 2. (**b**) The suppression of dV/dI(V) curves in normal magnetic field. Some dV/dI(V,B) asymmetry can also be seen for this sample. The data are obtained at 30 mK temperature.

We observe similar non-Ohmic dV/dI behavior for several Au-NiTe${}_{2}$ junctions, and the critical temperature of the non-linearity suppression is varied within the 150–200 mK range from sample to sample. Figure 4 represents one of the examples. dV/dI(V) non-linearity is qualitatively the same in Figure 4a as for the first junction in Figure 2a. Some dV/dI(V,B) asymmetry can also be seen for this sample in normal magnetic fields, as depicted in Figure 4b.

The drop in dV/dI(V) cannot be attributed to the usual scattering at the Au-NiTe${}_{2}$ interface, since the scattering should be described as the effective interface potential and, therefore, it always results in a wide dV/dI peak at zero bias [36]. On the other hand, the dV/dI(V) continuous increase to both sides of zero bias is known for electron–phonon or electron–magnon scattering [37,38], however, the temperature and bias voltage ranges apparently refer to much smaller energy scales in our experiment.

The observed behavior strongly resembles the known one for typical Andreev reflection at the NS interface between a normal metal and a superconductor [35,39]. In this case, multiple dV/dI(V) peaks in Figure 2a should be attributed to geometrical resonances in NSN junctions [40,41,42]. In the case of a thin superconducting layer (S), these resonances are known as crossed Andreev reflection, where an incident electron and a reflected hole appear on both sides of the S layer [43]. The resonance positions are determined by the superconducting gap, so they follow the gap temperature dependence in Figure 2b.

The standard BCS temperature dependence [35] is shown by the dashed lines in Figure 2b for several peak positions. The width of the central dV/dI drop corresponds to the temperatures of the dV/dI(V) curves smearing in Figure 2 and Figure 4. The claim on the superconductivity is also confirmed by the magnetic field suppression of the effect. For the planar experimental geometry, it is natural to have the critical field anisotropy for normal and in-plane field orientations in Figure 3a,b, respectively.

### 3.2. Double Nb-NiTe${}_{2}$-Nb Junctions

The claim on the interface superconductivity can be further supported by the supercurrent investigations between two Nb-NiTe${}_{2}$ interfaces. We study electron transport between two neighbor 1μm Nb leads in a standard two-point technique. All the wire resistances are excluded, which is necessary for low-impedance samples.

**Figure 5:**
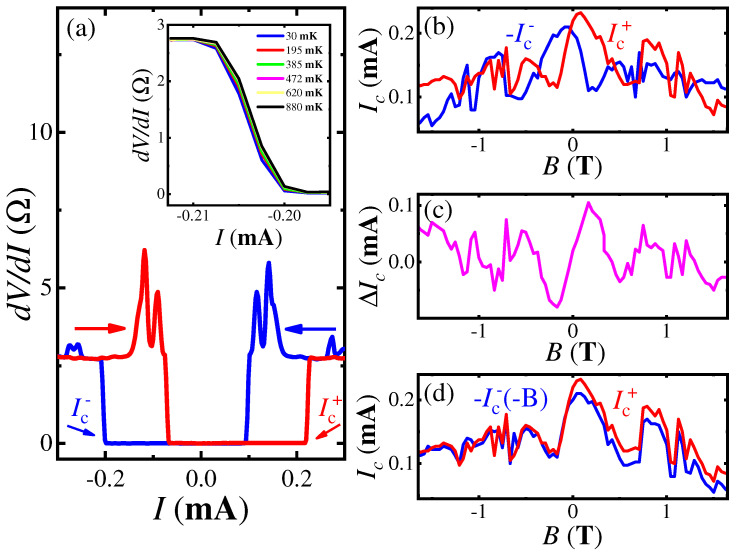
(Color online) Josephson diode effect for a double Nb-NiTe${}_{2}$-Nb junction. (**a**) Examples of dV/dI(V) curves for two different current sweep directions for 26 mT in-plane magnetic field. Apart from standard hysteresis for superconducting–resistive and resistive–superconducting transitions, non-reciprocal response is seen even for the same superconducting–resistive part of the curves, as depicted by $I_{c}^{+}=0.22$ mA and $I_{c}^{-}=-0.20$ mA values. The inset shows the temperature stability of the dV/dI(V) curves in the 30 mK–1.2 K range. (**b**) Critical currents $I_{c}^{+}\left(B\right)$ (red) and $-I_{c}^{-}\left(B\right)$ (blue) for two different current sweep directions, respectively, in dependence of the in-plane magnetic field. (**c**) The difference $\Delta I_{c}=I_{c}^{+}-I_{c}^{-}$, which is nearly antisymmetric in the magnetic field, with multiple sign changes [23]. (**d**) Another demonstration of field antisymmetry, as a coincidence of $I_{c}^{+}\left(B\right)$ and the reversed $-I_{c}^{-}\left(-B\right)$ curves, which also confirms the high accuracy of $I_{c}$ determination.

Figure 5a clearly demonstrates Josephson dV/dI(I) curves for two different current sweep directions. As expected, the zero-resistance state appears below some critical current $I_{c}$. The absolute value of $I_{c}$ is strongly different for resistive–superconducting and superconducting–resistive transitions, so dV/dI(I) curves show standard hysteresis with the current sweep direction. Furthermore, the transition region is nearly independent of temperature in the 30 mK–1.2 K range, because of the high critical temperature on niobium, see the inset to Figure 5a.

However, the absolute $I_{c}$ values are also different for two current sweep directions even for the same superconducting–resistive part of the curves, as depicted by $I_{c}^{+}=0.22$ mA and $I_{c}^{-}=-0.20$ mA values in Figure 5a for +26 mT in-plane magnetic field. This non-reciprocal response is known as the Josephson diode effect, the simplest manifestations of which is the direction dependence of the critical current.

To obtain $I_{c}$ with high accuracy at fixed *B*, we sweep the current ten times from the zero value (i.e., from the superconducting dV/dI = 0 state) to some value well above the $I_{c}$ (the resistive dV/dI>0 state) and then determine $I_{c}$ as an average value of dV/dI breakdown positions.

The result is presented in Figure 5b as $I_{c}^{+}$ (red) and $-I_{c}^{-}$ (blue) for two different current sweep directions, respectively. The difference $\Delta I_{c}=I_{c}^{+}-I_{c}^{-}$ is governed by magnetic field, demonstrating odd-type field dependence in Figure 5c, as expected for the Josephson diode effect. This can also be demonstrated by the direct comparison of $I_{c}^{+}\left(B\right)$ and $-I_{c}^{-}\left(-B\right)$ in Figure 5d. The curves coincide well, and they show a somewhat distorted Fraunhofer pattern [44,45]. The Josephson diode effect is due to the spin-momentum locking in topological Dirac semimetal NiTe${}_{2}$, which connects the superconductivity and the topological surface states.

## 4. Discussion

As a result, dV/dI(V) curves for an individual Au-NiTe${}_{2}$ interface strongly resemble the effect of the interface superconductivity in the NiTe${}_{2}$ Dirac semimetal. A similar result was previously observed [16,17] for another Dirac semimetal Cd${}_{3}$As${}_{2}$, which was attributed to the flat band formation at the Au-Cd${}_{3}$As${}_{2}$ interface [16]. Thus, the interface superconductivity should reflect the fundamental physics of topological Dirac semimetals, irrespective of the specifics of the particular material.

As an opposite example of material-dependent effects, bulk superconductivity is known for pressurized Te-deficient NiTe${}_{2}$ [46] as well as for doped [47] Cd${}_{3}$As${}_{2}$ single crystal samples. These effects can be ruled out in our experiment, since (i) bulk superconductivity is not observed for our NiTe${}_{2}$ crystals according to four-point resistance data in Figure 1c; (ii) X-ray spectroscopy reveals almost stoichiometric Ni${}_{1-x}$Te${}_{2}$ crystal with a slight Ni deficiency (*x* < 0.06); and (iii) there is no external pressure in our experiment.

On the other hand, interface superconductivity can appear due to the flat-band formation [4,5,6,7], which is the topological phenomenon [8,9,10]. In Dirac semimetals, strain generically acts as an effective gauge field on Dirac fermions and creates pseudo-Landau orbitals without breaking the time-reversal symmetry [48]. The zero-energy Landau orbitals form a flat band in the vicinity of the Dirac point, so the high density of states of this flat band may produce the interface superconductivity.

Strain can occur at the interface between Au and NiTe${}_{2}$ due to the lattice mismatch, or, more likely, due to the local bending of the crystal by thick electrodes. The strain-induced flat-band formation is predicted in pristine NiTe${}_{2}$ at ambient pressure [49], so the statement on the Au-NiTe${}_{2}$ interface superconductivity is quite reasonable. It is important, that we observe finite four-point resistance between different contacts in Figure 1c, which well correspond to the fact that strain-induced flat-band formation only occurs at the Au-NiTe${}_{2}$ interface.

In topological materials, the flat-band and topological surface states are closely connected: they appear due to the bulk-boundary correspondence, so the flat-band is the topological surface state at zero energy [5,9]. The topological surface states are essentially spin-polarized [21] due to the spin-momentum locking. This may result in the dV/dI(V,B) asymmetry, observed for Au-NiTe${}_{2}$ junctions subjected to the normal magnetic fields (see Figure 3a and Figure 4b). More importantly, spin-momentum locking is responsible for the Josephson diode effect. We wish to mention that there is no magnetic ordering in the bulk of NiTe${}_{2}$, since the direct magnetic measurements (by Lake Shore Cryotronics 8604 VSM magnetometer) reveal purely paramagnetic moment for our crystals in accordance with the previously reported data [33].

The Josephson diode effect appears as the direction-dependent Josephson current, where the direction of the Cooper pair momentum determines the polarity of the effect. In Figure 5, the finite Cooper pair momentum appears as antisymmetric $\Delta I_{c}\left(B\right)$ dependence. The finite momentum pairing results from the momentum shift of topological surface states of NiTe${}_{2}$ under an in-plane magnetic field, so the Josephson diode effect originates from spin-helical topological surface states, in an otherwise centrosymmetric system. Thus, the Josephson diode effect connects the superconductivity and the topological surface states in NiTe${}_{2}$.

As a result, topological surface states carry the Josephson current on the pristine NiTe${}_{2}$ surface in between the two neighbor metallic contacts, while these states are responsible for the flat-band formation at the Au-NiTe${}_{2}$ interface, and, therefore, the interface superconductivity.

## 5. Conclusions

In conclusion, we experimentally investigate charge transport through a single planar junction between a NiTe${}_{2}$ Dirac semimetal and a normal gold lead. At milli-Kelvin temperatures, we observe non-Ohmic dV/dI(V) behavior resembling Andreev reflection at a superconductor–normal metal interface, while NiTe${}_{2}$ bulk remains non-superconducting. We connect this behavior with interfacial superconductivity due to the flat-band formation at the Au-NiTe${}_{2}$ interface. Furthermore, we demonstrate the pronounced Josephson diode effect on the pristine NiTe${}_{2}$ surface, which results from the momentum shift of topological surface states under an in-plane magnetic field. This observation further supports the claim on the flat-band-induced superconductivity, since the flat-band and topological surface states are closely connected.

## Data Availability

The data are available from the authors upon reasonable request.
